# The Impact of the COVID-19 Pandemic on Women’s Perinatal Mental Health: Preliminary Data on the Risk of Perinatal Depression/Anxiety from a National Survey in Italy

**DOI:** 10.3390/ijerph192214822

**Published:** 2022-11-10

**Authors:** Laura Camoni, Fiorino Mirabella, Antonella Gigantesco, Sonia Brescianini, Maurizio Ferri, Gabriella Palumbo, Gemma Calamandrei, on behalf of the Perinatal Mental Health Network

**Affiliations:** Center for Behavioral Sciences and Mental Health, Italian National Health Institute, 00161 Rome, Italy

**Keywords:** perinatal depression, perinatal anxiety, screening, mental health

## Abstract

Increasing evidence suggests that during the COVID-19 pandemic, anxiety and depression during the perinatal period increased. The aim of the study is to estimate the prevalence of risk for both maternal depression and anxiety among women attending 18 healthcare centres in Italy during the SARS-COV-2 pandemic and to investigate the psychosocial risks and protective factors associated. It was divided into a retrospective phase (2019, 2020, and the first nine months of 2021) and a prospective phase (which began in November 2021 and it is still ongoing), which screened 12,479 and 2349 women, respectively, for a total of 14,828 women in the perinatal period. To evaluate the risk of anxiety and depression, the General Anxiety Disorder-7 (GAD-7), the Edinburgh Postnatal Depression Scale (EPDS), and an ad hoc form were used to collect sociodemographic variables. In the prospective study, the average age of the women is 31 (range 18–52) years. Results showed that the percentage of women who had EPDS score ≥9 increased from 11.6% in 2019 to 25.5% in the period ranging from November 2021 to April 2022. In logistic regression models, the variables associated with the risk of depression at a level ≤0.01 include having economic problems (OR 2.16) and not being able to rely on support from relatives or friends (OR 2.36). Having the professional status of the housewife is a lower risk (OR 0.52). Those associated with the risk of anxiety include being Italian (OR 2.97), having an education below secondary school level (OR 0.47), having some or many economic problems (OR 2.87), being unable to rely on support from relatives or friends (OR 2.48), and not having attended an antenatal course (OR 1.41). The data from this survey could be useful to determine the impact of the SARS-COV-2 pandemic on women and to establish a screening program with common and uniformly applied criteria which are consistent with national and international women’s mental health programs.

## 1. Introduction

The emergency triggered by the COVID-19 pandemic is impacting many aspects of society, including those related to mental well-being [1].

With the pandemic outbreak, several factors came into play that can affect a person’s psychological well-being: the presence of a concrete threat of illness to oneself or others, isolation, social distance, changes in daily routines, loss of work routines, and an excess of information that is often inaccurate and contradictory, all of which can increase insecurity, tension, and even anxiety and sadness [2]. Longitudinal studies conducted in different countries during the COVID-19 pandemic confirmed increased psychological distress associated with a reduction in social contacts, forced isolation, and quarantine [3,4,5,6]. Recent studies have also identified specific risk factors for mental health during the COVID-19 outbreak which, among others, include young age, female gender, low income, and having a pre-existing mental health condition [7]. In such an emergency, women in their perinatal period, which is a term the World Health Organization (WHO) [8] has used for the duration of pregnancy and the year after birth, represent a particularly vulnerable population as they are typically exposed to more significant risk factors regarding depression and anxiety [9,10,11]. The theoretical framework of this study is the biopsychosocial model of perinatal depression and anxiety that considers perinatal women as persons in their complexity and totality, where besides the biological, psychological, and social dimensions, there are also other factors that may affect their well-being or distress. This model, in fact, systematically uses biological, psychological, and social factors, including their complex interactions, in the understanding of psychophysical health and has been largely applied to women’s health in the perinatal period [12]. Several reasons may explain the increased vulnerability to depression and anxiety in women during and after pregnancy, including the physical, emotional, and hormonal changes associated with pregnancy and childbirth, as well as the impending life change associated with pregnancy, childbirth, and the redefinition of the family unit [12]. Many psychosocial factors can also affect women’s mental health during this period. Among these, unplanned pregnancy, partner relationship difficulties, previous mental disorders, low social support, low self-esteem, and work stress are all risk factors that can provoke prenatal anxiety and depression [13,14,15,16]. Studies that have analysed past epidemics have found that isolation causes concern and strong anxiety reactions, particularly in pregnant and postpartum women [17]. Quarantine, loss of routine, and social support can also negatively disturb new mothers and their babies. The lockdown policies have caused a drastic reduction in the physical presence of the parental and friend network, which constitutes a protective factor for mental health and, above all, for the risk of suicide [18,19]. All this, together with the widespread fear of COVID-19 infection, contributes to a state of anxiety and worry that might have had serious consequences on women’s mental health, especially in those most at risk [20,21,22]. It could be hypothesised that during the COVID-19 pandemic, social distancing measures in many countries created difficulty in accessing health services which, in many cases, prevented pregnant women from receiving the necessary support.

In Italy, in some health centres, during the initial phase of the pandemic, antenatal classes were officially suspended due to social distancing measures. Antenatal classes make it possible for women to relate to other women, share their state of mind, and receive peer support, all of which produce important benefits, a fact confirmed in international studies [23].

This study has two objectives: (i) to monitor the prevalence, identified through screening, of depression and anxiety risks among pregnant and postpartum women in Italy and to assess any changes that occurred during the COVID-19 pandemic; (ii) to evaluate the association between the risk of anxiety and depression in the perinatal period and sociodemographic, socioeconomic, and clinical variables. For aim (i), the hypothesis is that the pandemic has worsened the situation, and prevalence, identified through screening, of depression and anxiety, has increased. For aim (ii), the hypothesis is that a new pattern of psychosocial factors could determine the risk of developing depression and anxiety in the perinatal period.

To this aim, we involved a network of mother-child health centres and hospital services that performed screening of depression/anxiety of women in their perinatal period who have had access to these services since 2019.

## 2. Materials and Methods

### 2.1. Study Design

In September 2020, the Italian Perinatal Mental Health Network was established at the Reference Center for Behavioral Sciences and Mental Health of the Italian National Health Institute. This group, coordinated by the ISS, initiated the biennial Survey of Perinatal Mental Health during the SARS-COV-2 pandemic.

This cross-sectional survey is divided into a retrospective and a prospective phase. The retrospective phase collected aggregate data on women screened for depression during their perinatal period (i.e., from pregnancy to one year postpartum) in 2019, 2020, and the first nine months of 2021.

The prospective phase began in November 2021 and was based on the collection of individual data regarding the risk of anxiety and depression in women during pregnancy and up to 12 months in the postpartum period. Women who scored higher than the cut-off value were enrolled in a program for in-depth diagnosis and, after its completion, were invited to treatment and follow-up [24,25].

#### Data Collection Processes and Screening Tools

In the retrospective phase, referents of healthcare centres filled out an aggregate data collection form, while in the prospective phase, women accessing centres filled out self-reporting questionnaires on maternal depression, anxiety, and significant psychosocial risk factors. The aggregate data collection form includes:

1. Information on the number of women to whom the screening was offered;
2. The number of women who accepted screening;
3. The number of women who underwent screening;
4. The number of women who were positive/negative at screening.

The instrument used to assess depression risk was the Edinburgh Postnatal Depression Scale (EPDS) [26], the most widely used worldwide on account of its cross-cultural sensitivity and specificity. The Italian version was used, which was validated by Benvenuti [27], which has a high internal consistency (Cronbach’s alpha, 0.80). The EPDS is a self-assessment scale consisting of 10 items, answered on a 4-point Likert scale, which yields scores ranging from 0 to 3. The total score ranges from 0 to 30, with higher scores indicating more severe depressive symptoms. The items capture most symptoms of depression, such as hope for the future, depressed mood, guilt, anxiety, worry, sleep disturbance, and thoughts of self-harm.

The first two items are positively worded, and the remaining eight items are negatively worded. The choice of the cut-off value to be used depends on the goals of the assessment: a cut-off value of 9/10 seems most appropriate for a screening program or a community survey, while a cut-off value of 12/13 is usually recommended for clinical assessments and in research, especially in studies of effectiveness [28] in which treatment is to be given only to those who are more likely to have a depressive problem in the perinatal period. The cut-off score adopted in the present study was ≥9.

The GAD-7 was used for anxiety status assessment to assess the risk and severity of generalised anxiety disorder. It is a self-administered questionnaire consisting of 7 items. It is used to determine the frequency of anxiety symptoms in the past two weeks on a 4-point Likert scale ranging from 0 to 3 (not at all/sometimes/more than half the days/all days). Items include nervousness, inability to stop brooding, excessive brooding, restlessness, difficulty in relaxing, mild irritability, and fear that something terrible might happen. The total score ranges from 0 to 21 and is the sum of the scores of the individual items, with the following categories of symptom severity: 1–4 minimal symptoms; 5–9 mild symptoms; 10–14 moderate symptoms; 15–21 severe symptoms. A cut-off value ≥8 was considered optimal for sensitivity without compromising specificity [29].

In addition, an ad hoc form was created to collect some sociodemographic variables (age, educational level, working status, marital status, economic status), information about the pregnancy (whether the woman had had other pregnancies, whether she had resorted to assisted reproductive technology, previous pregnancies, abortions), information about previous depression problems and use of psychotropic drugs, and information about perceived family and social support (support from partner, friends, or relatives for practical help or psychological support when needed).

### 2.2. Sample

Eighteen healthcare centres (obstetrics and gynaecology wards, psychiatry hospital departments, and maternal-child health centres) (Table 1) in seven Italian regions already involved in the screening and care of women at risk of perinatal depression/anxiety participated in the study. Participation in the study was voluntary. Women were recruited during routine check-ups performed by each centre in the pre-or postpartum period, from the first stage of pregnancy to 12 months after childbirth. Women were screened only once during the study period.

Before participating in the study, women received oral and written information about the content and objectives of the study. The women willing to participate in the study were asked to sign the informed consent form and were able to withdraw from the study at any time. The study was approved by the Ethics Committee of the Italian National Health Institute.

### 2.3. Statistical Analysis

The data were analysed using the Statistical Package for Social Science (SPSS) version 28.0 for Windows. Standard descriptive statistics were calculated, and the chi-square statistical significance test was applied to assess the association between categorical variables and screening outcomes. Finally, odd ratios (OR) were estimated using stepwise logistic regression models, and the variables significantly associated with screening outcome in the univariate analysis with a significance ≤ 0.10 were included in the multivariate model.

## 3. Results

### 3.1. Depression Screening in the Period 2019–2022

Table 2 shows the screening data for the risk of depression as assessed by the EPDS test with a cut-off score ≥ 9. For the period 2019–2021, the aggregated data of the retrospective study were used. Of the 16,533 enrolled subjects, 12,479 performed the screening in the period 2019–2021.

**Figure 1:**
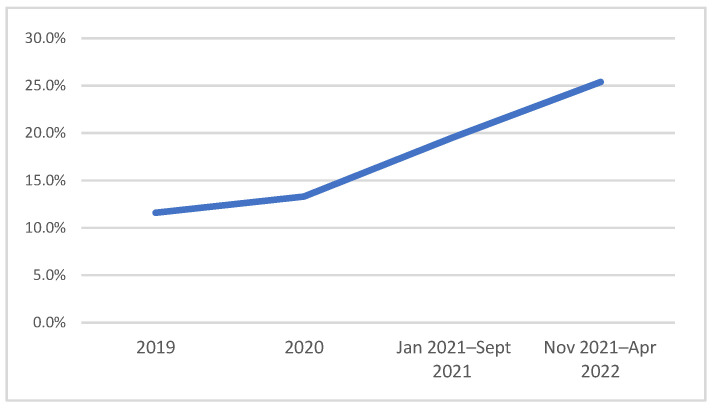
EPDS score ≥ 9 trend in the period 2019–2022.

The prospective study, on the other hand, used the individual data collected in the first 6 months, from November 2021 to April 2022. Of the 2456 enrolled subjects, 2349 completed the screening from November 2021 to April 2022. Table 2 reports the participation rate in different periods of the study. Women who had an EPDS score ≥9 totaled 11.6% in 2019, 13.3% in 2020, 19.5% in the period between January and September 2021, and 25.5% in the period between November 2021 and April 2022 (Figure 1). In this study, the EPDS showed good internal consistency with a Cronbach alpha of 0.84.

### 3.2. Associations between the Risk of Depression and Anxiety with Socio-Demographic Variables and Personal, Family, and Clinical History

The univariate analyses (Table 3) of the individual data relating to the period November 2021-April 2022 highlighted that women who had EPDS score ≥ 9 were more likely to be unemployed or temporarily employed, did not live with their partners, had severe economic problems, had had abortions in the past, did not attend an antenatal class, had little or no social and family support, used psychiatric drugs, had a history of depression or anxiety, and had a family member suffering from depression or anxiety.

Regarding the risk of anxiety, women who had GAD-7 score ≥ 8 were Italian, had a high school diploma or university degree, had a temporary job or were unemployed, did not live with a partner, had serious or some economic problems, had had abortions in the past, had not planned their pregnancy, had resorted to assisted reproductive technology, had not attended antenatal courses, had little or no social or family support, had used psychotropic drugs, had suffered from depression or anxiety in the past and had a family member suffering from depression or anxiety problems. The internal consistency of GAD-7 was 0.86 (Cronbach’s alpha).

### 3.3. Association of Sociodemographic, Socioeconomic Characteristics, Clinic Variables and Risk of Depression and Anxiety, Results of Multiple Logistic Regression Model

The multivariate analysis yielded a statistically valid (*p* < 0.001) and conceptually acceptable model for predicting depression (Table 4), which consisted of seven variables, and which guarantees the correct classification of 75% of the subjects. The variables found to be significant were as follows: having economic problems (OR 2.16), living alone (OR 2.82), not being able to rely on support from relatives or friends (OR 2.36), and not being able to rely on support from a partner (OR 1.61). Having the professional status of the housewife has a lower risk (OR 0.52).

Table 5 shows the statistically valid multivariate model (*p* < 0.001) of eleven variables, which guarantees the correct classification of 83.5% of subjects. The variables that showed a greater risk of anxiety were as follows: being Italian (OR 2.97), having a severe economic condition (OR 2.87) and average income (OR 1.34), being unable to rely on support from relatives or friends (OR 2.48), or a partner (OR 1.72), and not having attended an antenatal course (OR 1.41). It has been shown that a scholastic level below secondary school (OR 0.47) and high school (OR 0.74) has a lower risk of anxiety compared to women with higher levels of education.

Concerning clinical variables (Table 6), all three variables included in both models were significant for depression and anxiety, respectively, with an OR of 2.35 and 2.19 for women currently taking psychotropic drugs, an OR of 2.29 and 2.65 for women with a past diagnosis of depression or anxiety, and an OR of 1.82 and 1.84 for women with family members diagnosed with depression or anxiety in the past.

## 4. Discussion

These are the first Italian national data on the impact of the COVID-19 pandemic on mothers’ risk of depression and anxiety during the perinatal period, which involved more than 14,000 subjects who performed the screening during the period 2019–2022.

We hypothesised that the restriction and isolation measures introduced to contain the spread of the SARS-COV-2 virus and the concerns about possible infection [30], the uncertainty about the possible consequences of infection on the health of the unborn child, the limited obstetric monitoring, and the dilemma of vaccination, could have increased the natural vulnerability of women during this delicate phase of life.

In agreement, the prevalence of depressive symptomatology found in our sample appears to have been steadily increasing from 2019 to 2022 (Figure 1), with the value for the most recent year more than twice the value at the beginning of the survey. In the final year, the observed prevalence appears to be significantly higher than that observed in similar studies conducted in Italy before the pandemic period using a similar methodology [11,12,13,14,15,16,17,18,19,20,21,22,23,24,25,26,27,28], with values falling typically in the range of 10–13% [31].

However, it should be noted that this study used a particularly sensitive cut-off value for EPDS, which is also recommended in the Italian manual for this type of study [27]. The prevalence estimates reported in this study may therefore be higher than other studies reported in the literature that used higher cut-off values. The variables associated with the risk of depression and anxiety in the univariate analyses are consistent with those found in most international studies regarding risk factors in the perinatal period [11,32,33].

The psychological and social profile that emerges is that of a woman with a tendency towards anxiety and depression, a history of family and personal distress, low economic status, and low perceived social and family support. All the associated variables provide important clues, but the presence of spurious associations which frequently come out during these analyses may make it difficult to draw any definitive conclusions.

A true predictive model, which accounts for possible confounding factors, can only be obtained from a logistic regression analysis. From this, a theoretically acceptable and plausible picture emerges, composed of variables that are essentially consistent with those predominantly reported in the literature, and which are always oriented to indicate the biopsychosocial paradigm [34] as the most recognised reading code for depressive and anxiety-related problems associated with the perinatal period. Among these variables, lack of perceived social and family support and major economic difficulties stand out in terms of depression risk.

The perceived lack of support from the partner and social network exacerbates the natural insecurities faced by the new mother in caring for her child [35].

International studies of pandemics, and COVID-19 in particular, have shown significant economic impacts in both the short and long term [36]. Moreover, as surveys of the general population show, social isolation is associated with a range of negative psychological effects including anxiety, depression, and worries about real financial difficulties [37] which may persist in subsequent years [17]. Overall, these findings fit well with the conceptual framework describing biopsychosocial vulnerability and depression or anxiety in perinatal women [38]. Past relevant illness might contribute to the accumulation of life stressors that prevent effective adjustment of regulatory mechanisms and coping strategies. Previous studies that employed multivariate analyses to integrate variables such as stressful life events and history of mental health conditions suggested that women with specific mental health traits are more sensitive to the effects of adversity and stress events [39]. The relationship observed between depression and anxiety and lack of social support from family and friends was well documented in previous studies [40] and our findings confirm that relationship. Similarly, socioeconomic deprivation is another factor linked to stress vulnerability, as observed in previous studies conducted on low-income population samples, suggesting that women with socioeconomic difficulties exhibit higher levels of stress and depression. Therefore, our findings support the biopsychosocial model of perinatal depression as proposed by Milgrom and Martin [34] and provide clear indications regarding the importance of investigating psychosocial risk factors during the perinatal period to the prevention of first incidences of mental health problems.

The present findings are also consistent with previous studies [16,39,41,42] on the association between depression and anxiety in the perinatal period and the worry of not being able to meet the necessary costs required to raise a child.

Particularly striking is the significant reduction in risk in the category of housewives compared with women with jobs. This is possibly due to a greater sense of protection and security associated with staying at home during the pandemic period and not exposing themselves to the risk of infection [43]. The considerations made about the role of low economic status and perceived social and family support could still explain anxiety risk.

The increased risk of anxiety appears to be particularly relevant for women who did not (or could not because of the pandemic contingency) attend antenatal classes. In Italy, during the initial phase of the pandemic, antenatal classes were officially suspended due to social distancing measures. The opportunity to identify with other women, which occurs in antenatal courses, contributes to the “normalization” of their state of mind [44]. The climate of “peer support” that develops in the groups, the psychoeducational component of the topics covered, and the feeling of being supported by healthcare services during this delicate phase of life can produce important benefits for the women who participate in these courses, as international studies also show [23]. Similarly, the presence of family members and partners during the key moments of pregnancy, such as ultrasound examinations or routine check-ups, was restricted, leading to feelings of loneliness and an increase in anxiety and depressive symptoms [32]. Access to hospital services, in the first year of the pandemic was often limited to “emergencies” only, making it difficult to meet women’s needs. Telephone, video calls and text messaging have been able to partially compensate for this shortcoming [22]. However, it is important to remember that these forms of contact require “intentionality”, whereas, during non-emergency times, contact can be casual and when problems arise, as networks of family and friends (if available and able to act as support networks) can step in to provide support and assistance.

Women who have resorted to medically assisted technology also have an increased risk of anxiety, likely related to previous disappointments and fear of not being able to carry the current pregnancy to term [45].

Of note is the unique association between anxiety risk, Italian nationality, and high education level. Regarding foreign women participating in the screening, it can be assumed that those who accepted screening were better integrated, had a better knowledge of the Italian language, and had better access to health system services. As a consequence, this smaller sample of women (9.0% of the sample) could not possibly represent the population of foreign women, which are very often in precarious economic conditions and with difficulties in accessing available social/health services.

As for the association between higher education levels and anxiety, it must be considered that women with bachelor’s degrees tend to have their children later in life and thus they are likely at higher risk of obstetric complications and hospitalisation. In addition, more educated women might have a greater knowledge of possible complications that can occur during pregnancy, and this can in turn increase a woman’s anxiety level [46].

The clinical variables, including the use of psychotropic drugs and a previous diagnosis of an anxious/depressive disorder, are all highly associated with the risk of depression and anxiety, a fact that has been reported in numerous international papers [47].

This study has some limitations. The composition of our sample included centres that were not homogeneous regarding the risk of depression in the women or the unborn child; maternal–child health centres recruited mostly healthy women, whereas hospital departments admitted women with illnesses, high-risk pregnancies, or pregnant women whose newborns/infants were diagnosed with potential health problems before birth.

The study has the typical limitations of a cross-sectional study, which means it is impossible to infer the direction of causality of the associations between the variables studied and the risk of depression and anxiety. It should also be noted that the results of the study refer to screening the risk of depression or anxiety. For diagnostic confirmation, the results should be further investigated with appropriate procedures. In addition, the study did not collect any information on the characteristics of women who did not agree to participate in the screening.

Finally, all data collected from women in the study were self-reported, which could have caused possible assessment bias.

## 5. Conclusions

These data, which are part of a large national sample, highlight the adverse impact of the pandemic emergency on women’s mental health in the perinatal period, confirming the role of known psychosocial factors for anxiety and depression and their exacerbation during the two years of the COVID-19 pandemic. Subsequent analyses will provide information on annual trends, participating units, and particular subgroups of the population, for example, women with high-risk pregnancies compared to physiological pregnancies or to foreign women. Although still preliminary, these findings highlight the urgency of monitoring the psychological well-being of women in their perinatal period. The implementation of screening programs in the perinatal period would allow for the early identification of women at higher risk of anxiety/depression and thus their inclusion in effective intervention programs, thereby promoting the development of the mother–child relationship and mental health throughout life.

## Figures and Tables

**Table 1:** Total screening performed, number and percentage of EPDS score ≥ 9 according to the healthcare Centres involved in the study, 2019–2022.

Location	Name	Unit Type	*n*. Screening Performed	*n*. EPDS ≥ 9 (%)
Belluno	Feltre Hospital	Obstetrics and gynaecology Unit	2246	216 (9.6)
Bergamo	Local Health Authority Bergamo ovest	Maternal-child health centres	2206	301 (13.6)
Campobasso	Local Health Authority	Maternal-child health centres	500	41 (8.2)
Catania	Local Health Authority	Maternal-child health centres	406	57 (12.4)
Catania	ARNAS, Garibaldi Nesima Hospital	Obstetrics and gynaecology Unit	736	102 (13.9)
Enna	Enna Hospital	Obstetrics and gynaecology Unit	227	115 (50.7)
Foggia	Perinatal Depression Multicentric Observatory	Psychiatry hospital Department	688	170 (24.7)
Palermo	Buccheri La Ferla Hospital	Obstetrics and gynaecology Unit	1050	198 (18.9)
Palermo	Perinatal Depression Multicentric Observatory	Psychiatry Hospital Department	100	26 (26.0)
Pisa	Surgical Pathology department and University of Pisa	Department of Surgical, Medical and Molecular Pathology, and Critical Care Medicine	460	134 (29.1)
Rome	Perinatal Psychopathology Service, Sapienza University of Rome, Umberto I Hospital, Perinatal Depression Multicentric Observatory	Psychiatry Hospital Department	609	207 (34,0)
Rome	Cristo Re Hospital	Obstetrics and gynaecology Unit	2467	356 (14.4)
Rome	Bambino Gesù Hospital, Fetal and perinatal medicine and surgery *	Hospital Department	355	146 (41.1)
Rome	Perinatal Depression Multicentric Observatory, Tor Vergata University and Hospital	Psychiatry Hospital Department	60	44 (51.9)
Rome	Perinatal Depression Multicentric Observatory, A.Gemelli Hospital	Psychiatry Hospital Department	180	64 (35.6)
Treviso	Local Health Authority *	Maternal-child health centres	32	2 (6.3)
Vicenza	Maternal and Paternal Perinatal Disorder Service	Local Psychiatry Department	156	81 (51.9)
Viterbo	Perinatal Depression Multicentric Observatory, Peripartum Clinic	Psychiatry Hospital Department	2296	238 (10.4)
Total			14,828	2498 (16.8)

* Did not participate in the retrospective study.

**Table 2:** Depression screening (EPDS score ≥ 9) in the period 2019–2022.

	2019		2020		January 2021– September 2021		November 2021– April 2022	
	*n*	%	*n*	%	*n*	%	*n*	%
Proposals for participation in the study	5118		4780		6635		2456	
Agreements to participate in the study	4240	82.8	4481	93.7	5203	78.4	2392	97.4
Disagreements to participate in the study	878	17.2	299	6.3	1432	21.6	64	2.6
Perform the screening	3362	79.3	4279	95.3	4838	93.0	2349	98.2
Not perform the screening	878	20.7	202	4.7	365	7.00	43	1.8
EPDS < 9	2971	88.4	3712	86.7	3895	80.5	1752	74.6
EPDS ≥ 9	391	11.6	567	13.3	943	19.5	597	25.4

**Table 3:** Associations between the risk of depression and anxiety with sociodemographic variables and personal, family, and clinical history.

	*n*	% EPDS ≥ 9	*p*	*n*	% GAD-7 ≥ 8	*p*
Age						
<30	583	26.6	0.44	581	17.2	0.70
30–35	927	26.1		924	16.5	
>35	779	23.9		778	18.0	
Nationality						
Italian	2101	25.6	0.99	2095	17.8	0.04
Non-Italian	211	25.6		211	12.3	
Educational level						
Primary or illiterate	28	28.6	0.34	29	13.8	0.08
Secondary school	277	20.9		274	11.7	
High School	992	26.0		990	17.6	
Degree	1039	25.9		1037	18.1	
Occupational status						
Housewife	367	19.1	0.001	367	12.0	0.009
Student, unemployed	386	30.3		381	19.7	
Temporary employee	181	31.5		182	22.0	
Permanent employee	1391	24.7		1389	16.9	
Marital status						
Single	183	31.1	0.12	183	19.7	0.55
Separated, divorced, or widowed	38	18.4		37	13.5	
Married or cohabiting	2114	25.0		2109	17.0	
Family situation						
Lives alone/with others/parent	30	56.7	0.001	27	33.3	0.002
Lives with partner	2244	24.8		2241	16.7	
Economic status						
Some or many problems	191	42.9	0.001	189	30.7	0.001
A few problems	1294	25.2		1290	16.5	
Average to high status	821	21.3		821	14.7	
Previous pregnancies						
Yes	1249	25.2	0.94	1081	16.5	0.43
No	1085	25.3		1246	17.7	
Past abortion(s)						
Yes	730	27.3	0.08	727	19.3	0.05
No	1584	23.9		1581	15.9	
Children living at the time of this pregnancy/birth						
Yes	1038	24.2	0.34	1036	16.0	0.22
No	1270	25.9		1264	18.0	
Planned pregnancy						
Yes	1626	24.1	0.24	1624	15.4	0.004
No	658	26.4		654	20.3	
Resort to assisted reproductive technology						
Yes	197	27.9	0.36	197	22.3	0.04
No	2106	24.9		2099	16.7	
Participation in antenatal classes						
Yes	828	22.7	0.042	824	14.8	0.02
No	1473	26.5		1471	18.6	
Rely on support from relatives or friends						
No support at all	48	41.7	0.001	48	27.1	0.001
Not enough support	255	43.1		254	31.9	
Enough support	822	25.2		821	16.2	
More than enough support	1210	21.0		1205	14.3	
Rely on support from partner						
No support at all	29	51.7	0.001	29	37.9	0.001
Not enough support	92	43.5		92	33.7	
Enough support	457	27.6		457	18.4	
More than enough support	1753	23.1		1746	15.4	
Current use of psychotropic drugs						
Yes	45	62.2	0.001	45	51.1	0.001
No	2282	24.4		2275	16.4	
Previous diagnosis of depression or anxiety						
Yes	276	46.4	0.001	273	36.6	0.001
No	2059	22.4		2055	14.5	
Family member with a previous diagnosis of depression or anxiety						
Yes	364	39.3	0.001	364	28.6	0.001
No	1970	22.6		1963	14.9	

**Table 4:** Association of sociodemographic and socioeconomic characteristics and risk of depression (EPDS score ≥ 9): results of multiple logistic regression model.

	OR	CI 95% OR	*p*
Occupational status			
housewife	0.52	0.38–0.72	0.001
Student, unemployed	1.00	0.75–1.33	0.997
Temporary employee	1.10	0.76–1.60	0.606
Permanent employee	1		
Economic status			
Some or many problems	2.16	1.45–3.21	0.001
A few problems	1.24	0.99–1.56	0.062
Average high status	1		
Family situation			
Lives alone/with others/parents	2.82	1.22–6.56	0.016
Lives with partner	1		
Rely on support from relatives or friends			
not at all/not enough support	2.36	1.73–3.24	0.001
Enough support	1.19	0.94–1.52	0.148
More than enough support	1		
Rely on support from partner			
not at all/not enough support	1.61	1.0–2.58	0.05
Enough support	1.11	0.84–1.45	0.473
More than enough support	1		
Past abortion(s)			
Yes	1		
No	0.88	0.71–1.10	0.261
Participation in antenatal classes			
Yes	1		
No	1.16	0.94–1.44	0.176

**Table 5:** Association of sociodemographic and socioeconomic characteristics and risk of anxiety (GAD-7 score ≥ 8): results of multiple logistic regression model.

	OR	CI 95% OR	*p*
Nationality			
Italian	2.97	1.67–5.27	0.001
Non-Italian	1		
Educational level			
Illiterate, primary, secondary school	0.47	0.29–0.78	0.003
High School	0.74	0.57–0.98	0.033
Degree	1		
Occupational status			
Housewife	0.69	0.45–1.05	0.084
Student, unemployed	1.15	0.81–1.64	0.422
Temporary employee	1.22	0.80–1.86	0.357
Permanent employee	1		
Economic status			
Some or many problems	2.87	1.79–4.62	0.001
A few problems	1.34	1.01–1.79	0.044
Average to high status	1		
Family situation			
Lives alone/with others/parents	1.55	0.59–4.08	0.378
Lives with partner	1		
Rely on support from relatives or friends			
not at all/not enough support	2.48	1.73–3.56	0.001
Enough support	1.09	0.81–1.46	0.566
More than enough support	1		
Rely on support from partner			
not at all/not enough support	1.72	1.01–2.92	0.045
Enough support	1.19	0.87–1.64	0.279
More than enough support	1		
Planned pregnancy			
Yes	1		
No	1.31	1.00–1.71	0.049
Participation in antenatal classes			
Yes	1		
No	1.41	1.09–1.83	0.009
Past abortion(s)			
Yes	1		
No	0.91	0.70–1.17	0.465
Resort to assisted reproductive technology			
Yes	1		
No	0.63	0.42–0.94	0.023

**Table 6:** Association of clinic variables and risk of depression (EPDS score ≥ 9) and anxiety (GAD-7 score ≥ 8): results of multiple logistic regression model.

	EPDS ≥ 9			GAD-7 ≥ 8		
	OR	CI 95% OR	*p*	OR	CI 95% OR	*p*
Current use of psychotropic drugs						
No	1			1		
Yes	2.35	1.22–4.54	0.011	2.19	1.14–4.19	0.019
Previous diagnosis of depression or anxiety						
No	1			1		
Yes	2.29	1.73–3.04	<001	2.65	1.95–3.59	<001
Family member with a previous diagnosis of depression or anxiety						
No	1			1		
Yes	1.82	1.42–2.34	<001	1.84	1.40–2.42	<001

## Data Availability

Not applicable.

## Appendix A

Perinatal Mental Health Network: Franca Aceti, Servizio di psicopatologia perinatale, Sapienza Università di Roma, Policlinico Umberto I- Osservatorio Multicentrico per la Depressione Perinatale; Ilaria Adulti, Osservatorio Multicentrico per la Depressione Perinatale, Università di Tor Vergata, Roma; Lucia Aite, Ospedale Pediatrico Bambino Gesù, Unità Operativa di Psicologia Clinica e diagnosi prenatale, Roma; Piero Bagolan, Ospedale Pediatrico Bambino Gesù, Unità Operativa di Psicologia Clinica e diagnosi prenatale, Roma; Gina Barbano, UOC Infanzia Adolescenza Famiglia e Consultori, Distretto Treviso nord—sede di Oderzo, Azienda ULSS 2 Marca trevigiana; Antonello Bellomo Osservatorio Multicentrico Depressione Perinatale, UOC Psichiatria Foggia; Silvia Bucci, Ospedale Pediatrico Bambino Gesù, Unità Operativa di Psicologia Clinica e diagnosi prenatale, Roma; Simona Cappelletti, Ospedale Pediatrico Bambino Gesù, Unità Operativa di Psicologia Clinica e diagnosi prenatale, Roma; Marina Cattaneo, Consultorio familiare di Treviglio, ASST Bergamo Ovest; Elda Cengia, ULSS 1 Dolomiti—Ospedale di Feltre Reparto Ostetricia e Ginecologia, Belluno; Monica Del Sole, Osservatorio Multicentrico Depressione Perinatale, Ambulatorio Peripartum Viterbo; Angela Fabiano Divisione Materno Infantile ASP Catania; Chiara Falamesca, Ospedale Pediatrico Bambino Gesù, Unità Operativa di Psicologia Clinica e diagnosi prenatale, Roma; Laura Favretti, ULSS 1 Dolomiti—Ospedale di Feltre Reparto Ostetricia e Ginecologia, Belluno; Laura Ferraro, Osservatorio Multicentrico Depressione Perinatale, UOC Psichiatria Palermo; Nicoletta Giacchetti, Servizio di psicopatologia perinatale Sapienza Università di Roma, Policlinico Umberto I, Osservatorio Multicentrico per la Depressione Perinatale; Antonella Grillo, Divisione Materno Infantile ASP Catania; Teresa Grimaldi Capitello, Ospedale Pediatrico Bambino Gesù, Unità Operativa di Psicologia Clinica e diagnosi prenatale, Roma; Chiara Ionio, Università Cattolica Sacro Cuore, Milano; Daniele La Barbera, Osservatorio Multicentrico Depressione Perinatale, UOC Psichiatria Palermo; Marta Landoni Università Cattolica Sacro Cuore, Milano; Angelo Marcheggiani, Consultorio di Campobasso; Marianna Mazza, Osservatorio Multicentrico Depressione Perinatale, Dipartimento di Psichiatria Policlinico Gemelli; Loredana Messina, Ospedale Buccheri La Ferla, Palermo; Cinzia Niolu, Osservatorio Multicentrico per la depressione perinatale, Università di Tor Vergata, Roma; Giovanna Picciano, Consultorio di Campobasso; Maria Pistillo, UOC Ostetricia e Ginecologia, ASP Enna; Laura Raho, Ospedale Pediatrico Bambino Gesù, Unità Operativa di Psicologia Clinica e diagnosi prenatale, Roma; Miryam Regonesi, Consultorio familiare di Treviglio, ASST Bergamo Ovest; Rossana Riolo Ambulatorio genitori senza depressione, ALSS 8-Berica; Angela Rossi, Ospedale Pediatrico Bambino Gesù, Unità Operativa di Psicologia Clinica e diagnosi prenatale, Roma; Gabriele Sani, Osservatorio Multicentrico Depressione Perinatale, Dipartimento di Psichiatria Policlinico Gemelli, Roma; Martina Smorti, Dipartimento di Patologia Chirurgica, Medica, Molecolare e dell’Area Critica, Università di Pisa; Damiana Tomasello, ARNAS, PO Garibaldi Nesima, Catania; Antonella Triggiani, Ospedale Cristo Re, Roma.
